# Acknowledgment of Reviewers

**DOI:** 10.1265/ehpm.26-00069

**Published:** 2026-04-15

The Editor-in-Chief and the Editorial Board of *Environmental Health and Preventive Medicine* would like to express their sincere appreciation to the following reviewers for their time, expertise, and valuable contributions to the peer-review process in 2025.

The high quality of the journal depends on the dedication of these scholars, and we are deeply grateful for their support.

Yuji Shimizu
Sumitaka Kobayashi
Takehiro Michikawa
Yu Cao
Kouji H. Harada
Keigo Saeki
Masayuki Shima
Hiroki Takada
Koki Kosami
Atsushi Mizukoshi
Masashi Ishizu
Hatasu Kobayashi
Tommaso Filippini
Masaru Miyao
Takamitsu Miyayama
Hiroaki Arima
Ichiro Fukunaga
Masanori Ogawa
Tomoki Shiozawa
Hisashi Adachi
Ryoji Hirota
Cimi Ilmiawati
Nobufumi Yasuda
Kei Asayama
Yuki Fujiwara
Yuki Ito
Nobumichi Kobayashi
Kohki Nakane
Kazunari Onishi
George Kuttiparichel Varghese
Takashi Yamauchi
Hiroshi Yatsuya
Ling-Chu Chien
Weijing Du
Akira Fujiyoshi
Toshiaki Hitomi
Mano Horinaka
Takuji Kishimoto
Naoyuki Kurokawa
Chan Lu
Akiko Matsumoto
Kenta Matsumura
Roger Nlandu Nlandu
Nobutaka Ohgami
Toshiyuki Onoda
Kenzo Takahashi
Yasunobu Aoki
Zheng-Guo Cui
Yoshiharu Fukuda
Yayoi Funakoshi
Jun Hata
Qiang He
Yusuke Hiraku
Atsuko Ikeda
Nahomi Imaeda
Sri Irianti
Midori Ishikawa
Hiroyoshi Iwata
Tetsuhiro Kidokoro
Chihaya Koriyama
Yohei Mineharu
Nathan Mise
Utako Murai
Chikage Nagano
Chikako Nakama
Harunobu Nakamura
Tasuku Okui
Koya Suzuki
Yoshiaki Tai
Mitsuo Uchida
Hiroo Wada
Laith N. Al-Eitan
Htoo Wai Aung
Kenichi Azuma
Kanae Bekki
Jamie M. Bogle
Chaiwut Bournoew
Roger Brown
Hao Chen
Wan-Ju Cheng
Chi-Tsun Chiu
Desdiani Desdiani
Shotaro Doki
Akifumi Eguchi
Orna Epel
Yukiko Fujii
Yoshihisa Fujino
Yuki Fujita
Kota Fukai
Federico Girolametti
Takahito Hayashi
Aya Hirata
Sahoko Ichihara
Takeyasu Kakamu
Siriporn Kamsa-Ard
Masashi Kato
Eiki Kimura
Yosuke Kimura
Fumiya Kinoshita
Katsuyasu Kouda
Hisaka Kurita
Yuquan Lu
Hengliang Lv
Nataša Marčun-Varda
Yasuyuki Matsuura
Francesco Meneguzzo
Marina Minami
Nobuyuki Miyai
Hiroyuki Miyauchi
Akihiko Nishikimi
Kyoko Nomura
Kazuyuki Okamura
Dayo Rotimi Omotoso
Atsushi Ono
Yuichiro Otsuka
Hongbin Peng
Rebecca Pratiti
Nicholas Ralston
Masaya Saito
Kiyomi Sakata
Bojan Simovski
Takahiro Tabuchi
Keiko Tanaka
Kazuhide Tezuka
Taisuke Tomonaga
Takashi Toyama
Yu-Hwei Tseng
Kayo Ueda
Shahbaz Ahmad Zakki
Cai Zong
Azham Umar Abidin
Yumi Abiko
Yuri Akamatsu
Hazim Alhiti
Hajime Ando
Yuria Asao
Seungho Baek
Willem I.J. Boer
Xiaoling Cai
Jingsi Chen
K. D. Datkhile
Hanifa M Denny
Somayeh Derakhshan
Keita Ebisu
Mathias Abiodun Emokpae
Xiaolu Feng
Tomoya Fujie
Maki Furutani
Shobhan Gaddameedhi
Wenjing Gao
Majid Ghayour-Mobarhan
Masahiro Hashizume
Daisuke Hori
Hyogo Horiguchi
Masahisa Horiuchi
Menglong Hu
Masayoshi Ichiba
Yuzuru Iizuka
Hideki Imai
Hidekuni Inadera
Kazue Ishitsuka
Ken-Ichi Iwasaki
Runze Jiang
Yaping Jin
Toshiyuki Kaji
Atif Kamal
Kei Kamide
Michihiro Kamijima
Şeyma Aliye Kara
Kota Katanoda
Tsuguhiko Kato
Eman Omar Khashaba
Kosuke Kiyohara
Yoshiki Kuroda
Yuki Kuwabara
Keisuke Kuwahara
Qing Li
Jufen Liu
Eri Maeda
Manal Mahmoud
Genxiang Mao
Bind Marie-Abèle
Dragan Mijakoski
Yoshikazu Mikami
Jun Miyata
Wataru Miyazaki
Emi Morita
Satoshi Nakai
Chris Fook Sheng Ng
Nlandu R. Ngatu
Toshirou Nishida
Midori Nishiyama
Yasuhiro Noda
Shingo Noguchi
Tomoko Oguri
Tetsuya Ohira
Masayuki Okuda
Mina Ostovari
Marina Ruxandra Otelea
Asuka Oyama
Isao Oze
Makiko Sekiyama
Ken Shirato
Katsumi Shozugawa
Branislava Stojković
Xin Su
Harumitsu Suzuki
Junta Tagusari
Masumi Takada
Midori Takada
Raquel Teixeira
Masaharu Tsubokura
Takashi Tsuji
Akihiko Uchiyama
Osamu Udagawa
Jun Ueyama
Ichiro Wakabayashi
Li Wang
Wei Wang
Hao Xiang
Yi Xing
Siddika Songül Yalçın
Mitsuya Yamakita
Tadashi Yamashita
Noboru Yamashita
Hyeran Yang
Atiye Seda Yar
Takahiko Yoshida
Kouichi Yoshimasu
Hiroki Yoshioka

(The reviewers are listed in descending order based on the number of reviews completed.)

Kouji H. HARADA

Editor-in-Chief

*Environmental Health and Preventive Medicine*

